# 2,2-Dimethyl-*N*,*N*′-bis­(4-nitro­benzyl­idene)propane-1,3-diamine

**DOI:** 10.1107/S1600536808037033

**Published:** 2008-11-13

**Authors:** Hoong-Kun Fun, Hadi Kargar, Reza Kia

**Affiliations:** a X-ray Crystallography Unit, School of Physics, Universiti Sains Malaysia, 11800 USM, Penang, Malaysia; b Department of Chemistry, School of Science, Payame Noor University (PNU), Ardakan, Yazd, Iran

## Abstract

In the title compound, C_19_H_20_N_4_O_4_, a potential bidentate Schiff base ligand, each imino (C=N) functional group is coplanar with the adjacent benzene ring. The two benzene rings form a dihedral angle of 10.52 (6)°. Inter­molecular C—H⋯O contacts link neighbouring mol­ecules into supra­molecular array with an *R* ^2^_2_(32) ring motif and a C—H⋯π contact is also present.

## Related literature

For details of hydrogen-bond motifs, see: Bernstein *et al.* (1995 ▶). For related structures, see: Li *et al.* (2005 ▶); Bomfim *et al.* (2005 ▶); Glidewell *et al.* (2005 ▶, 2006 ▶); Sun *et al.* (2004 ▶); Fun *et al.* (2008*a* ▶,*b* ▶).
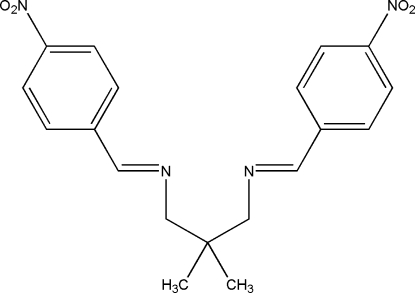


## Experimental

### 

#### Crystal data

C_19_H_20_N_4_O_4_
*M* *_r_* = 368.39
Monoclinic, 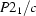

*a* = 7.8219 (1) Å
*b* = 19.9716 (4) Å
*c* = 12.0125 (3) Å
β = 92.831 (1)°
*V* = 1874.25 (6) Å^3^
*Z* = 4
Mo *K*α radiation
μ = 0.09 mm^−1^
*T* = 100 (1) K
0.45 × 0.19 × 0.08 mm

#### Data collection

Bruker SMART APEXII CCD area-detector diffractometer
Absorption correction: multi-scan (**SADABS**; Bruker, 2005 ▶) *T*_min_ = 0.959, *T*_max_ = 0.993
21845 measured reflections
6784 independent reflections
4307 reflections with *I* > 2σ(*I*)
*R*_int_ = 0.048

#### Refinement

*R*[*F* ^2^ > 2σ(*F* ^2^)] = 0.061
*wR*(*F* ^2^) = 0.152
*S* = 1.04
6784 reflections
244 parameters
H-atom parameters constrained
Δρ_max_ = 0.36 e Å^−3^
Δρ_min_ = −0.23 e Å^−3^

### 

Data collection: *APEX2* (Bruker, 2005 ▶); cell refinement: *SAINT* (Bruker, 2005 ▶); data reduction: *SAINT*; program(s) used to solve structure: *SHELXTL* (Sheldrick, 2008 ▶); program(s) used to refine structure: *SHELXTL*; molecular graphics: *SHELXTL*; software used to prepare material for publication: *SHELXTL* and *PLATON* (Spek, 2003 ▶).

## Supplementary Material

Crystal structure: contains datablocks global, I. DOI: 10.1107/S1600536808037033/tk2324sup1.cif

Structure factors: contains datablocks I. DOI: 10.1107/S1600536808037033/tk2324Isup2.hkl

Additional supplementary materials:  crystallographic information; 3D view; checkCIF report

## Figures and Tables

**Table 1:** Hydrogen-bond geometry (Å, °)

*D*—H⋯*A*	*D*—H	H⋯*A*	*D*⋯*A*	*D*—H⋯*A*
C1—H1*A*⋯O4^i^	0.93	2.52	3.4330 (18)	168
C17—H17*A*⋯O2^ii^	0.93	2.48	3.4063 (18)	171
C19—H19*A*⋯*Cg*1^iii^	0.96	2.86	3.8058 (16)	171

Symmetry codes: (i) 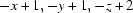; (ii) 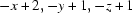; (iii) 
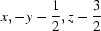
. *Cg*1 is the centroid of the C12–C17 benzene ring.

**Fig. 1.**
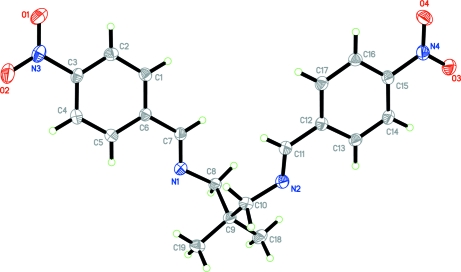
The molecular structure of (I) with atom labels and 50% displacement ellipsoids for non-H atoms.

**Fig. 2.**
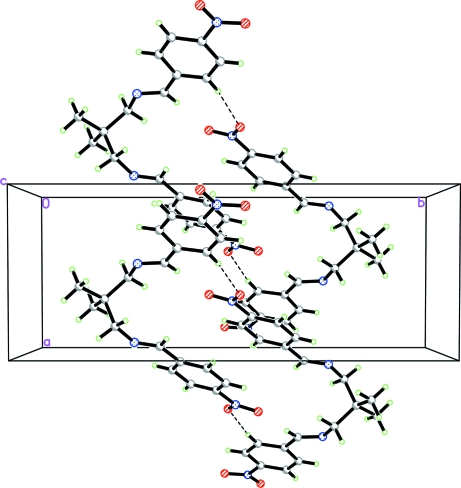
The crystal packing of (I), viewed down the c-axis showing the linking of neighbouring molecules via R22(32) ring motifs. Intermolecular hydrogen bonds are shown as dashed lines.

## supplementary crystallographic information

### Comment

Schiff bases are one of most prevalent mixed-donor ligands in the field of coordination chemistry. They play an important role in the development of coordination chemistry related to catalysis and enzymatic reactions, magnetism, and supramolecular architectures. Structures of Schiff bases derived from substituted benzaldehydes and closely related to the title compound, (I), have been reported previously (Li *et al.*, 2005; Bomfim *et al.*, 2005; Glidewell *et al.*, 2005, 2006; Sun *et al.*, 2004; Fun *et al.*, 2008*a*,*b*).

Each imino (C ═N) functional group in (I), Fig. 1, is co-planar with the adjacent benzene ring. Intramolecular C—H···O contacts form five-membered rings, producing *S*(5) ring motifs (Bernstein *et al.*, 1995). The two benzene rings form a dihedral angle of 10.52 (6)°. Intermolecular C—H···O contacts link neighbouring molecules into supramolecular array with *R^2^_2_(32)* ring motif, Fig. 2 and Table 1. The crystal structure is further stabilized by weak C—H···π interactions, ( Table 1.

### Experimental

The synthetic method has been described earlier (Fun *et al.*, 2008). Single crystals suitable for X-ray diffraction were obtained by evaporation of an ethanol solution of (I) held at room temperature.

### Refinement

All hydrogen atoms were positioned geometrically and refined using a riding model with C—H = 0.93 - 0.97 Å, and with *U*_iso_(H) = 1.2-1.5*U*_eq_(C).

### Crystal data

**Table:**

C_19_H_20_N_4_O_4_	*F*_000_ = 776
*M**_r_* = 368.39	*D*_x_ = 1.306 Mg m^−^^3^
Monoclinic, *P*2_1_/*c*	Mo *K*α radiation λ = 0.71073 Å
Hall symbol: -P 2ybc	Cell parameters from 3284 reflections
*a* = 7.8219 (1) Å	θ = 2.7–28.9º
*b* = 19.9716 (4) Å	µ = 0.09 mm^−^^1^
*c* = 12.0125 (3) Å	*T* = 100 (1) K
β = 92.831 (1)º	Block, colourless
*V* = 1874.25 (6) Å^3^	0.45 × 0.19 × 0.08 mm
*Z* = 4	

### Data collection

**Table:**

Bruker SMART APEXII CCD area-detector diffractometer	6784 independent reflections
Radiation source: fine-focus sealed tube	4307 reflections with *I* > 2σ(*I*)
Monochromator: graphite	*R*_int_ = 0.048
*T* = 100(1) K	θ_max_ = 32.6º
φ and ω scans	θ_min_ = 2.0º
Absorption correction: multi-scan(*SADABS*; Bruker, 2005)	*h* = −11→11
*T*_min_ = 0.959, *T*_max_ = 0.993	*k* = −30→24
21845 measured reflections	*l* = −16→18

### Refinement

**Table:**

Refinement on *F*^2^	Secondary atom site location: difference Fourier map
Least-squares matrix: full	Hydrogen site location: inferred from neighbouring sites
*R*[*F*^2^ > 2σ(*F*^2^)] = 0.061	H-atom parameters constrained
*wR*(*F*^2^) = 0.152	*w* = 1/[σ^2^(*F*_o_^2^) + (0.063*P*)^2^ + 0.2454*P*] where *P* = (*F*_o_^2^ + 2*F*_c_^2^)/3
*S* = 1.04	(Δ/σ)_max_ < 0.001
6784 reflections	Δρ_max_ = 0.36 e Å^−^^3^
244 parameters	Δρ_min_ = −0.23 e Å^−^^3^
Primary atom site location: structure-invariant direct methods	Extinction correction: none

### Special details

**Table:**

Experimental. The low-temperature data was collected with the Oxford Cyrosystem Cobra low-temperature attachment.
Geometry. All e.s.d.'s (except the e.s.d. in the dihedral angle between two l.s. planes) are estimated using the full covariance matrix. The cell e.s.d.'s are taken into account individually in the estimation of e.s.d.'s in distances, angles and torsion angles; correlations between e.s.d.'s in cell parameters are only used when they are defined by crystal symmetry. An approximate (isotropic) treatment of cell e.s.d.'s is used for estimating e.s.d.'s involving l.s. planes.
Refinement. Refinement of *F*^2^ against ALL reflections. The weighted *R*-factor *wR* and goodness of fit *S* are based on *F*^2^, conventional *R*-factors *R* are based on *F*, with *F* set to zero for negative *F*^2^. The threshold expression of *F*^2^ > σ(*F*^2^) is used only for calculating *R*-factors(gt) *etc*. and is not relevant to the choice of reflections for refinement. *R*-factors based on *F*^2^ are statistically about twice as large as those based on *F*, and *R*- factors based on ALL data will be even larger.

### Fractional atomic coordinates and isotropic or equivalent isotropic displacement parameters (Å^2^)

**Table:**

	*x*	*y*	*z*	*U*_iso_*/*U*_eq_	
O1	1.36590 (14)	0.55914 (6)	0.45685 (10)	0.0335 (3)	
O2	1.36634 (15)	0.48567 (6)	0.32431 (10)	0.0376 (3)	
O3	0.04816 (14)	0.42804 (6)	1.17606 (9)	0.0317 (3)	
O4	0.13096 (14)	0.52456 (5)	1.11560 (10)	0.0313 (3)	
N1	0.91963 (15)	0.26572 (6)	0.65265 (11)	0.0229 (3)	
N2	0.44695 (14)	0.27677 (6)	0.73309 (11)	0.0232 (3)	
N3	1.32746 (15)	0.50399 (7)	0.41710 (12)	0.0263 (3)	
N4	0.12292 (14)	0.46308 (6)	1.10932 (10)	0.0240 (3)	
C1	1.07594 (17)	0.43692 (7)	0.64720 (12)	0.0214 (3)	
H1A	1.0348	0.4512	0.7146	0.026*	
C2	1.17251 (17)	0.48038 (7)	0.58500 (12)	0.0222 (3)	
H2A	1.1983	0.5234	0.6103	0.027*	
C3	1.22894 (16)	0.45767 (7)	0.48441 (12)	0.0212 (3)	
C4	1.19437 (18)	0.39416 (8)	0.44352 (13)	0.0254 (3)	
H4A	1.2337	0.3806	0.3753	0.031*	
C5	1.09965 (17)	0.35135 (7)	0.50692 (13)	0.0245 (3)	
H5A	1.0751	0.3083	0.4813	0.029*	
C6	1.04054 (16)	0.37232 (7)	0.60928 (12)	0.0195 (3)	
C7	0.94133 (16)	0.32672 (7)	0.67822 (12)	0.0205 (3)	
H7A	0.8936	0.3432	0.7421	0.025*	
C8	0.81845 (17)	0.22410 (7)	0.72454 (12)	0.0220 (3)	
H8A	0.7833	0.2506	0.7871	0.026*	
H8B	0.8885	0.1874	0.7537	0.026*	
C9	0.65824 (17)	0.19558 (7)	0.66089 (12)	0.0211 (3)	
C10	0.52727 (18)	0.25158 (7)	0.63434 (13)	0.0228 (3)	
H10A	0.5843	0.2883	0.5986	0.027*	
H10B	0.4391	0.2346	0.5821	0.027*	
C11	0.44070 (16)	0.33980 (7)	0.74535 (12)	0.0211 (3)	
H11A	0.4901	0.3673	0.6933	0.025*	
C12	0.35718 (16)	0.37059 (7)	0.83998 (12)	0.0192 (3)	
C13	0.25521 (16)	0.33267 (7)	0.90909 (12)	0.0214 (3)	
H13A	0.2384	0.2873	0.8946	0.026*	
C14	0.17916 (17)	0.36202 (7)	0.99872 (12)	0.0222 (3)	
H14A	0.1125	0.3369	1.0453	0.027*	
C15	0.20531 (16)	0.43004 (7)	1.01696 (12)	0.0203 (3)	
C16	0.30321 (16)	0.46938 (7)	0.94913 (12)	0.0210 (3)	
H16A	0.3173	0.5149	0.9627	0.025*	
C17	0.37936 (17)	0.43876 (7)	0.86057 (12)	0.0215 (3)	
H17A	0.4461	0.4641	0.8143	0.026*	
C18	0.58134 (19)	0.14167 (8)	0.73376 (14)	0.0289 (3)	
H18A	0.4811	0.1232	0.6959	0.043*	
H18B	0.5507	0.1611	0.8031	0.043*	
H18C	0.6639	0.1068	0.7481	0.043*	
C19	0.70671 (19)	0.16493 (7)	0.54990 (13)	0.0259 (3)	
H19A	0.6059	0.1477	0.5111	0.039*	
H19B	0.7870	0.1292	0.5638	0.039*	
H19C	0.7577	0.1987	0.5052	0.039*	

### Atomic displacement parameters (Å^2^)

**Table:**

	*U*^11^	*U*^22^	*U*^33^	*U*^12^	*U*^13^	*U*^23^
O1	0.0318 (6)	0.0268 (6)	0.0423 (7)	−0.0069 (5)	0.0043 (5)	0.0051 (5)
O2	0.0377 (6)	0.0447 (7)	0.0314 (7)	−0.0070 (5)	0.0128 (5)	0.0041 (5)
O3	0.0332 (6)	0.0354 (6)	0.0276 (6)	−0.0072 (5)	0.0108 (5)	−0.0019 (5)
O4	0.0381 (6)	0.0230 (6)	0.0334 (7)	0.0044 (4)	0.0082 (5)	−0.0026 (5)
N1	0.0213 (5)	0.0232 (6)	0.0244 (7)	−0.0026 (4)	0.0020 (5)	−0.0003 (5)
N2	0.0211 (5)	0.0225 (6)	0.0261 (7)	0.0004 (4)	0.0023 (5)	−0.0012 (5)
N3	0.0185 (5)	0.0297 (7)	0.0308 (7)	−0.0009 (5)	0.0019 (5)	0.0075 (6)
N4	0.0202 (5)	0.0280 (7)	0.0238 (7)	0.0009 (5)	0.0024 (5)	−0.0016 (5)
C1	0.0197 (6)	0.0234 (7)	0.0211 (7)	0.0017 (5)	0.0010 (5)	−0.0015 (6)
C2	0.0197 (6)	0.0212 (7)	0.0255 (8)	−0.0003 (5)	−0.0016 (5)	−0.0005 (6)
C3	0.0162 (6)	0.0235 (7)	0.0240 (7)	0.0008 (5)	0.0021 (5)	0.0055 (6)
C4	0.0255 (7)	0.0286 (8)	0.0229 (8)	−0.0007 (6)	0.0076 (6)	−0.0018 (6)
C5	0.0254 (7)	0.0228 (7)	0.0257 (8)	−0.0011 (5)	0.0051 (6)	−0.0037 (6)
C6	0.0173 (6)	0.0204 (6)	0.0207 (7)	0.0001 (5)	0.0011 (5)	0.0007 (5)
C7	0.0187 (6)	0.0234 (7)	0.0196 (7)	−0.0003 (5)	0.0018 (5)	−0.0004 (6)
C8	0.0239 (6)	0.0208 (7)	0.0215 (7)	−0.0013 (5)	0.0020 (5)	0.0012 (6)
C9	0.0218 (6)	0.0177 (6)	0.0238 (7)	−0.0015 (5)	0.0013 (5)	−0.0003 (6)
C10	0.0241 (6)	0.0216 (7)	0.0227 (8)	0.0002 (5)	0.0002 (5)	−0.0019 (6)
C11	0.0195 (6)	0.0219 (7)	0.0220 (7)	−0.0001 (5)	0.0018 (5)	0.0010 (6)
C12	0.0170 (6)	0.0200 (6)	0.0204 (7)	0.0021 (5)	−0.0001 (5)	0.0015 (5)
C13	0.0195 (6)	0.0179 (6)	0.0268 (8)	−0.0007 (5)	0.0015 (5)	0.0002 (6)
C14	0.0192 (6)	0.0229 (7)	0.0248 (8)	0.0000 (5)	0.0036 (5)	0.0046 (6)
C15	0.0180 (6)	0.0228 (7)	0.0201 (7)	0.0024 (5)	0.0010 (5)	−0.0002 (5)
C16	0.0207 (6)	0.0180 (6)	0.0242 (7)	0.0002 (5)	0.0007 (5)	0.0003 (6)
C17	0.0207 (6)	0.0211 (7)	0.0229 (7)	−0.0005 (5)	0.0027 (5)	0.0031 (6)
C18	0.0282 (7)	0.0228 (7)	0.0357 (9)	−0.0041 (6)	0.0025 (6)	0.0048 (7)
C19	0.0275 (7)	0.0212 (7)	0.0289 (8)	0.0011 (5)	−0.0008 (6)	−0.0045 (6)

### Geometric parameters (Å, °)

**Table:**

O1—N3	1.2319 (17)	C8—H8B	0.9700
O2—N3	1.2256 (17)	C9—C18	1.530 (2)
O3—N4	1.2330 (15)	C9—C19	1.532 (2)
O4—N4	1.2316 (16)	C9—C10	1.539 (2)
N1—C7	1.2657 (18)	C10—H10A	0.9700
N1—C8	1.4597 (17)	C10—H10B	0.9700
N2—C11	1.2687 (18)	C11—C12	1.4732 (19)
N2—C10	1.4591 (18)	C11—H11A	0.9300
N3—C3	1.4714 (18)	C12—C17	1.3931 (19)
N4—C15	1.4671 (18)	C12—C13	1.4014 (18)
C1—C6	1.3914 (19)	C13—C14	1.3857 (19)
C1—C2	1.3921 (19)	C13—H13A	0.9300
C1—H1A	0.9300	C14—C15	1.389 (2)
C2—C3	1.383 (2)	C14—H14A	0.9300
C2—H2A	0.9300	C15—C16	1.3890 (18)
C3—C4	1.382 (2)	C16—C17	1.3868 (19)
C4—C5	1.3840 (19)	C16—H16A	0.9300
C4—H4A	0.9300	C17—H17A	0.9300
C5—C6	1.3990 (19)	C18—H18A	0.9600
C5—H5A	0.9300	C18—H18B	0.9600
C6—C7	1.4768 (18)	C18—H18C	0.9600
C7—H7A	0.9300	C19—H19A	0.9600
C8—C9	1.544 (2)	C19—H19B	0.9600
C8—H8A	0.9700	C19—H19C	0.9600
			
C7—N1—C8	118.34 (12)	C10—C9—C8	110.49 (11)
C11—N2—C10	117.20 (12)	N2—C10—C9	113.01 (12)
O2—N3—O1	123.55 (13)	N2—C10—H10A	109.0
O2—N3—C3	118.19 (13)	C9—C10—H10A	109.0
O1—N3—C3	118.26 (13)	N2—C10—H10B	109.0
O4—N4—O3	123.39 (12)	C9—C10—H10B	109.0
O4—N4—C15	118.14 (12)	H10A—C10—H10B	107.8
O3—N4—C15	118.47 (12)	N2—C11—C12	121.65 (13)
C6—C1—C2	120.46 (13)	N2—C11—H11A	119.2
C6—C1—H1A	119.8	C12—C11—H11A	119.2
C2—C1—H1A	119.8	C17—C12—C13	119.50 (12)
C3—C2—C1	118.00 (13)	C17—C12—C11	119.26 (12)
C3—C2—H2A	121.0	C13—C12—C11	121.24 (12)
C1—C2—H2A	121.0	C14—C13—C12	120.69 (13)
C4—C3—C2	123.10 (13)	C14—C13—H13A	119.7
C4—C3—N3	118.70 (13)	C12—C13—H13A	119.7
C2—C3—N3	118.18 (13)	C13—C14—C15	118.07 (12)
C3—C4—C5	118.16 (13)	C13—C14—H14A	121.0
C3—C4—H4A	120.9	C15—C14—H14A	121.0
C5—C4—H4A	120.9	C16—C15—C14	122.80 (13)
C4—C5—C6	120.52 (14)	C16—C15—N4	117.69 (12)
C4—C5—H5A	119.7	C14—C15—N4	119.48 (12)
C6—C5—H5A	119.7	C17—C16—C15	118.07 (13)
C1—C6—C5	119.75 (13)	C17—C16—H16A	121.0
C1—C6—C7	119.33 (12)	C15—C16—H16A	121.0
C5—C6—C7	120.92 (13)	C16—C17—C12	120.85 (12)
N1—C7—C6	121.69 (13)	C16—C17—H17A	119.6
N1—C7—H7A	119.2	C12—C17—H17A	119.6
C6—C7—H7A	119.2	C9—C18—H18A	109.5
N1—C8—C9	111.56 (12)	C9—C18—H18B	109.5
N1—C8—H8A	109.3	H18A—C18—H18B	109.5
C9—C8—H8A	109.3	C9—C18—H18C	109.5
N1—C8—H8B	109.3	H18A—C18—H18C	109.5
C9—C8—H8B	109.3	H18B—C18—H18C	109.5
H8A—C8—H8B	108.0	C9—C19—H19A	109.5
C18—C9—C19	109.76 (12)	C9—C19—H19B	109.5
C18—C9—C10	110.70 (11)	H19A—C19—H19B	109.5
C19—C9—C10	107.51 (12)	C9—C19—H19C	109.5
C18—C9—C8	107.98 (12)	H19A—C19—H19C	109.5
C19—C9—C8	110.41 (11)	H19B—C19—H19C	109.5
			
C6—C1—C2—C3	1.0 (2)	C11—N2—C10—C9	131.95 (13)
C1—C2—C3—C4	−0.2 (2)	C18—C9—C10—N2	49.59 (16)
C1—C2—C3—N3	178.53 (12)	C19—C9—C10—N2	169.47 (11)
O2—N3—C3—C4	4.2 (2)	C8—C9—C10—N2	−69.98 (14)
O1—N3—C3—C4	−176.02 (13)	C10—N2—C11—C12	177.85 (12)
O2—N3—C3—C2	−174.58 (13)	N2—C11—C12—C17	168.88 (14)
O1—N3—C3—C2	5.17 (19)	N2—C11—C12—C13	−11.5 (2)
C2—C3—C4—C5	−0.4 (2)	C17—C12—C13—C14	−1.2 (2)
N3—C3—C4—C5	−179.16 (13)	C11—C12—C13—C14	179.15 (13)
C3—C4—C5—C6	0.2 (2)	C12—C13—C14—C15	0.8 (2)
C2—C1—C6—C5	−1.2 (2)	C13—C14—C15—C16	0.3 (2)
C2—C1—C6—C7	178.54 (12)	C13—C14—C15—N4	178.38 (13)
C4—C5—C6—C1	0.6 (2)	O4—N4—C15—C16	6.84 (19)
C4—C5—C6—C7	−179.19 (13)	O3—N4—C15—C16	−173.73 (12)
C8—N1—C7—C6	−179.39 (12)	O4—N4—C15—C14	−171.35 (13)
C1—C6—C7—N1	−173.22 (14)	O3—N4—C15—C14	8.09 (19)
C5—C6—C7—N1	6.5 (2)	C14—C15—C16—C17	−0.9 (2)
C7—N1—C8—C9	118.97 (14)	N4—C15—C16—C17	−179.01 (12)
N1—C8—C9—C18	168.26 (11)	C15—C16—C17—C12	0.4 (2)
N1—C8—C9—C19	48.27 (15)	C13—C12—C17—C16	0.6 (2)
N1—C8—C9—C10	−70.53 (14)	C11—C12—C17—C16	−179.76 (13)

### Hydrogen-bond geometry (Å, °)

**Table:**

*D*—H···*A*	*D*—H	H···*A*	*D*···*A*	*D*—H···*A*
C1—H1A···O4^i^	0.93	2.52	3.4330 (18)	168
C17—H17A···O2^ii^	0.93	2.48	3.4063 (18)	171
C19—H19A···Cg1^iii^	0.96	2.86	3.8058 (16)	171

Symmetry codes: (i) −*x*+1, −*y*+1, −*z*+2; (ii) −*x*+2, −*y*+1, −*z*+1; (iii) *x*, −*y*−1/2, *z*−3/2.

## References

[bb1] Bernstein, J., Davis, R. E., Shimoni, L. & Chamg, N.-L. (1995). *Angew. Chem. Int. Ed. Engl.* **34**, 1555–1573.

[bb2] Bomfim, J. A. S., Wardell, J. L., Low, J. N., Skakle, J. M. S. & Glidewell, C. (2005). *Acta Cryst.* C**61**, o53–o56. 10.1107/S0108270104030719 15640596

[bb3] Bruker (2005). *APEX2*, *SAINT* and *SADABS* Bruker AXS Inc., Madison, Wisconsin, USA.

[bb4] Fun, H.-K., Kargar, H. & Kia, R. (2008*a*). *Acta Cryst.* E**64**, o1308. 10.1107/S1600536808018345 PMC2961864 21202936

[bb7] Fun, H.-K., Kargar, H. & Kia, R. (2008*b*). *Acta Cryst.* E**64**, o2273. 10.1107/S1600536808035307 PMC2960008 21581254

[bb5] Glidewell, C., Low, J. N., Skakle, J. M. S. & Wardell, J. L. (2005). *Acta Cryst.* E**61**, o3551–o3553. 10.1107/S0108768105004234 15772456

[bb6] Glidewell, C., Low, J. N., Skakle, J. M. S. & Wardell, J. L. (2006). *Acta Cryst.* C**62**, o1–o4. 10.1107/S0108270105035766 16397316

[bb8] Li, Y.-G., Zhu, H.-L., Chen, X.-Z. & Song, Y. (2005). *Acta Cryst.* E**61**, o4156–o4157.

[bb9] Sheldrick, G. M. (2008). *Acta Cryst.* A**64**, 112–122. 10.1107/S0108767307043930 18156677

[bb10] Spek, A. L. (2003). *J. Appl. Cryst.* **36**, 7–13.

[bb11] Sun, Y.-X., You, Z.-L. & Zhu, H.-L. (2004). *Acta Cryst.* E**60**, o1707–o1708.

